# Equine rhinitis B viruses in horse fecal samples from the Middle East

**DOI:** 10.1186/s12985-016-0547-x

**Published:** 2016-06-07

**Authors:** Patrick C. Y. Woo, Susanna K. P. Lau, Garnet K. Y. Choi, Yi Huang, Renate Wernery, Sunitha Joseph, Emily Y. M. Wong, Shyna K. Elizabeth, Nissy Annie Georgy Patteril, Tong Li, Ulrich Wernery, Kwok-Yung Yuen

**Affiliations:** State Key Laboratory of Emerging Infectious Diseases, The University of Hong Kong, Hong Kong, China; Department of Microbiology, The University of Hong Kong, Hong Kong, China; Research Centre of Infection and Immunology, The University of Hong Kong, Hong Kong, China; Carol Yu Centre for Infection, The University of Hong Kong, Hong Kong, China; Central Veterinary Research Laboratory, Dubai, United Arab Emirates

**Keywords:** Animal RNA viruses, Picornavirus, Horses

## Abstract

**Background:**

Among all known picornaviruses, only two species, equine rhinitis A virus and equine rhinitis B virus (ERBV) are known to infect horses, causing respiratory infections. No reports have described the detection of ERBV in fecal samples of horses and no complete genome sequences of ERBV3 are available.

**Methods:**

We performed a molecular epidemiology study to detect ERBVs in horses from Dubai and Hong Kong. Complete genome sequencing of the ERBVs as well as viral loads and genome, phylogenetic and evolutionary analysis were performed on the positive samples.

**Results:**

ERBV was detected in four (13.8 %) of the 29 fecal samples in horses from Dubai, with viral loads 8.28 × 10^3^ to 5.83 × 10^4^ copies per ml, but none of the 47 fecal samples in horses from Hong Kong by RT-PCR. Complete genome sequencing and phylogenetic analysis showed that three of the four strains were ERBV3 and one was ERBV2. The major difference between the genomes of ERBV3 and those of ERBV1 and ERBV2 lied in the amino acid sequences of their VP1 proteins. The Ka/Ks ratios of all the coding regions in the ERBV3 genomes were all <0.1, suggesting that ERBV3 were stably evolving in horses. Using the uncorrelated lognormal distributed relaxed clock model on VP1 gene, the date of the most recent common ancestor (MRCA) of ERBV3 was estimated to be 1785 (HPDs, 1176 to 1937) and the MRCA dates of ERBV1 and ERBV2 were estimated to be 1848 (HPDs, 1466 to 1949) respectively.

**Conclusions:**

Both acid stable (ERBV3) and acid labile (ERBV2) ERBVs could be found in fecal samples of horses. Detection of ERBVs in fecal samples would have implications for their transmission and potential role in gastrointestinal diseases as well as fecal sampling as an alternative method of identifying infected horses.

## Background

Picornaviruses are widely distributed in human and various animals in which they can cause respiratory, cardiac, hepatic, neurological, mucocutaneous and systemic diseases of varying severity [1]. Based on genotypic and serological characterization, the family *Picornaviridae* is currently divided into 26 genera. Among all the known picornaviruses, only two species, namely equine rhinitis A virus (ERAV) and equine rhinitis B virus (ERBV) are known to infect horses. ERAV, formerly called equine rhinovirus 1, belongs to the genus *Aphthovirus*; whereas ERBV, further subclassified into three serotypes, including ERBV1 and ERBV2 which are acid labile and were previously known as equine rhinovirus 2 and 3 respectively, and ERBV3 which was previously called acid-stable equine picornavirus, is the only species of a recently created species of the genus *Erbovirus* [2].

Both ERAV and ERBV are associated with respiratory diseases in horses and are therefore primarily found in nasal, nasopharyngeal and oral secretions [3–6]. Infected horses develop fever, anorexia, nasal discharge, cough and lymphadenitis. These respiratory diseases in horses are of particular importance because of their effect on the performance horse industry and they are also an economic burden for the horse owners. Sometimes ERAV can also be detected in plasma and urine [7, 8]. Although one study has reported the isolation of ERAV from the fecal samples of 13 out of 290 horses more than 50 years ago [9], so far no reports have described the detection of ERBV in fecal samples of horses. Since it is well-known that some respiratory picornaviruses, such as rhinoviruses [10], can be detected in fecal samples of infected individuals, we hypothesize that ERBV may be detectable in fecal samples of horses. This would have implications for transmission of the viruses, their potential role in gastrointestinal diseases and fecal sampling as an alternative method of identifying infected animals. To test this hypothesis, we performed a molecular epidemiology study on horses from Dubai and Hong Kong, two cities with the most popular horse racing industries. Comparative genomics of the complete genomes of ERBVs observed in fecal samples of the present study and the only two previously published complete genomes of ERBV1 and ERBV2 isolated from the nasal samples of horses were also performed.

## Results

### Horse surveillance and identification of ERBV

**Fig. 1:**
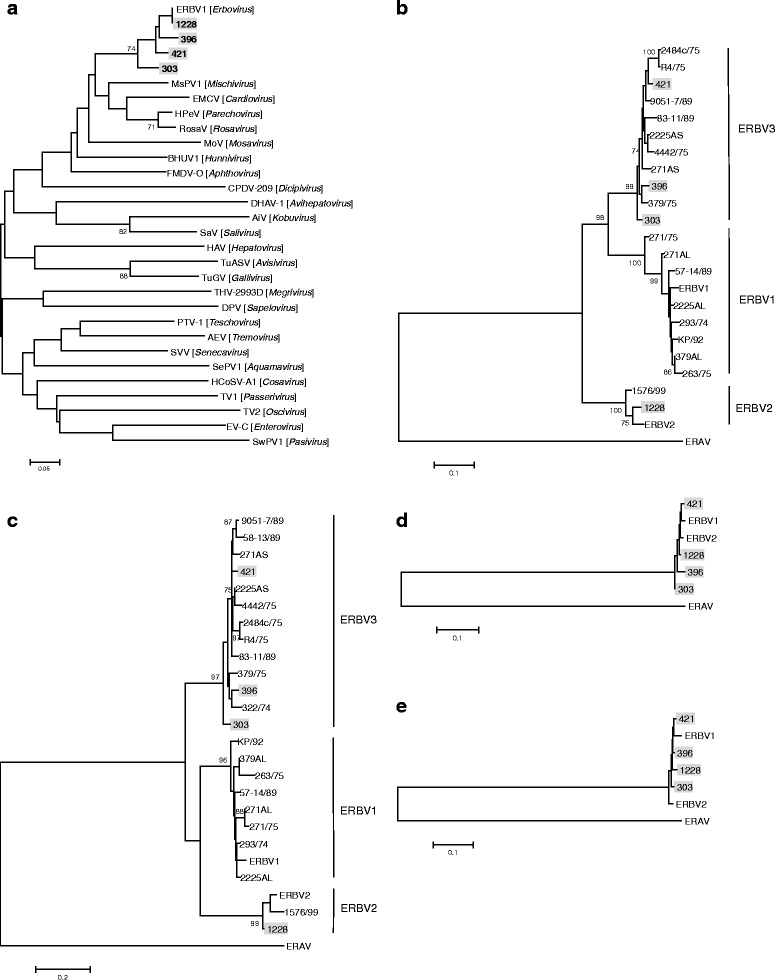
**a** Phylogenetic analysis of nucleotide sequences of the 73-bp fragment (excluding primer sequences) of the partial 5’ UTR of the ERBV detected from four fecal samples of horses in the present study. The four strains with genomes completely sequenced are shaded in gray. The trees were constructed by the neighbor-joining method, and bootstrap values calculated from 1000 trees. Bootstrap values expressed as percentages are shown at nodes and only those >70 % are shown. The scale bar indicates the estimated number of substitutions per 20 nucleotides. Phylogenetic analyses of the (**b**) P1, (**c**) VP1, (**d**) P2, and (**e**) P3 regions of the four ERBVs (shaded in gray) in the present study and those of other ERBVs. ERAV was included as the outgroup. The scale bars indicate the estimated number of substitutions per 10 (P1), 5 (VP1) and 10 (P2) and 10 (P3) amino acids, respectively. Virus abbreviations (GenBank accession numbers shown in parentheses): AEV, avian encephalomyelitis virus (NC_003990); AiV, Aichi virus A (NC_001918); BHUV1, Hunnivirus A (NC_018668); CPDV-209, Cadicivirus A (JN819202); DHAV-1, duck hepatitis A virus 1 (NC_008250); DPV, avian sapelovirus (NC_006553); EMCV, encephalomyocarditis virus (NC_001479); ERAV, equine rhinitis A virus (NC_003982); ERBV strains, 1576/99 (AY606998), 2225AS (GU799328), 2484c/75 (DQ108385), 263/75 (AY606990), 271AL (GU799329), 271AS (GU799330), 271/75 (AY606991), 293/74 (AY606988), 322/74 (AY606989), 379AL (GU799331), 379/75 (AY606992), 4442/75 (DQ108383), 57-14/89 (AY606994), 58-13/89 (AY606995), 83-11/89 (AY606996), 9051-7/89 (AY606997), KP/92 (AY606993), R4/75 (DQ108384); ERBV1, equine rhinitis B virus 1 (NC_003983); ERBV2, equine rhinitis B virus 2 (AF361253); EV-C, Enterovirus C (NC_002058); FMDV-O, foot-and-mouth disease virus type O (NC_004004); HAV, hepatitis A virus (NC_001489); HCoSV-A1, Cosavirus A (NC_012800); HPeV, human parechovirus (NC_001897); MsPV1, Mischivirus A (JQ814851); MoV, Mosavirus A (NC_023987); PTV-1, porcine teschovirus (NC_003985); RosaV, Rosavirus A (JF973686); SaV, Salivirus A (NC_012957); SePV1, Aquamavirus A (NC_009891); SwPV1, Swine pasivirus 1 (NC_018226); SVV, Seneca Valley virus (NC_011349); TV1, Passerivirus A (NC_014411); TV2, Oscivirus A (NC_014412); TuASV, Avisivirus A (KC465954); TuGV, Gallivirus A (NC_018400); THV-2993D, Megrivirus A (HM751199)

RT-PCR for a 111-bp fragment in the 5’-UTR of picornaviruses was positive in specimens from the fecal samples of four (13.8 %) of 29 horses from Dubai. The sequences from these positive samples had 89–95 % and 90-94 % nucleotide identities to the corresponding parts of the 5’-UTR in ERBV1 and ERBV2 respectively, suggesting the presence of ERBV in fecal samples of horses (Fig. 1a). On the other hand, none of the 47 fecal samples of horses from Hong Kong was positive.

### Real-time quantitative RT-PCR

Real-time quantitative RT-PCR showed that the amount of ERBV RNA in the four positive samples ranged from 8.28 × 10^3^ to 5.83 × 10^4^ copies per ml of fecal sample (Table 1).

**Table 1:** Viral load of ERBV in fecal samples

Sample number	Specimen collection date	Serotype	Concentration of ERBV (copies/ml)
303	April 2013	ERBV3	5.48 × 10^4^
396	June 2013	ERBV3	1.22 × 10^4^
421	June 2013	ERBV3	5.83 × 10^4^
1228	May 2014	ERBV2	8.28 × 10^3^

### Genome organization and coding potential

The complete genomes of the four strains of ERBVs (strains 303, 396, 421 and 1228) were sequenced and assembled. The size of the four genomes ranged from 8823 to 8831 bases, after excluding the polyadenylated tract; and the G + C content ranged from 49.5 to 50.3 % (Table 2). Each genome contained a large open reading frame of 7752 (strain 1228) or 7764 (strains 303, 396 and 421) bases, which encoded potential polyprotein precursors of 2584 (strain 1228) or 2588 (strains 303, 396 and 421) amino acids. The genome organization was similar to other picornaviruses, with the characteristic gene order 5’-VP4, VP2, VP3, VP1, 2A, 2B, 2C, 3A, 3B, 3C^pro^, 3D^pol^-3’. The hypothetical protease cleavage sites of the polyproteins, as determined by multiple alignments with the only two available complete genomes of ERBV1 and ERBV2, were analyzed (Table 3). The amino acids of strain 1228 at all the putative cleavage sites were identical to those of the two genomes of ERBV1 and ERBV2, whereas those of strains 303, 396 and 421 at all the cleavage sites, except VP1/2A, were identical to those in the two ERBV1 and ERBV2 genomes [11, 12].

**Table 2:** Comparison of genomic features of the four strains of ERBVs in the present study and ERBV1 and ERBV2 and the amino acid identities between their predicted P1, VP1, P2 and P3

Virus	Accession no.	Genome features		Pairwise amino acid identity (%)															
		Size	G + C	Strain 303				Strain 396				Strain 421				Strain 1228			
		(bases)	content	P1	VP1	P2	P3	P1	VP1	P2	P3	P1	VP1	P2	P3	P1	VP1	P2	P3
ERBV1	NC_003983	8828	0.49	78.0	69.4	97.4	96.8	78.1	69.8	96.6	97.4	77.7	69.8	98.1	97.6	73.0	66.7	97.9	96.3
ERBV2	AF361253	8821	0.50	74.8	63.3	98.1	97.7	74.9	63.8	97.4	97.5	74.3	63.4	98.1	97.0	95.3	95.3	98.4	96.7
ERBV3^a^	DQ108385	-	-	93.3	88.2	-	-	94.3	91.6	-	-	95.3	92.6	-	-	74.7	62.3	-	-
Strain 303	KX260138	8828	0.50	-	-	-	-	94.2	88.5	97.9	98.7	94.4	90.1	98.1	98.1	75.7	64.2	98.7	97.8
Strain 396	KX260139	8831	0.50	94.2	88.5	97.9	98.7	-	-	-	-	95.2	92.6	97.6	98.6	75.1	63.5	97.9	98.2
Strain 421	KX260140	8830	0.50	94.4	90.1	98.1	98.1	95.2	92.6	97.6	98.6	-	-	-	-	75.0	64.0	98.2	97.5
Strain 1228	KX260141	8823	0.50	75.7	64.2	98.7	97.8	75.1	63.5	97.9	98.2	75.0	64.0	98.2	97.5	-	-	-	-

^a^Only P1 region is available

**Table 3:** Coding potential of four strains of ERBVs in the present study

Putative proteins	Strain 303	Strain 396	Strain 421	Strain 1228
L	M^1^-R^219^	M^1^-R^219^	M^1^-R^219^	M^1^-R^219^
VP4	G^220^-L^290^	G^220^-L^290^	G^220^-L^290^	G^220^-L^289^
VP2	D^291^-E^546^	D^291^-E^546^	D^291^-E^546^	D^290^-E^545^
VP3	G^547^-E^775^	G^547^-E^775^	G^547^-E^775^	G^546^-E^775^
VP1	G^776^-S^1098^	G^776^-S^1098^	G^776^-S^1098^	G^776^-T^1094^
2A	N^1099^-G^1114^	N^1099^-G^1114^	N^1099^-G^1114^	N^1095^-G^1110^
2B	P^1115^-E^1397^	P^1115^-E^1397^	P^1115^-E^1397^	P^1111^-E^1393^
2C	G^1398^-Q^1714^	G^1398^-Q^17^14	G^1398^-Q^1714^	G^1394^-Q^1710^
3A	S^1715^-S^1847^	S^1715^-S^1847^	S^1715^-S^1847^	S^1711^-S^1843^
3B	R^1848^-E^1868^	R^1848^-E^1868^	R^1848^-E^1868^	R^1844^-E^1864^
3C	N^1869^-Q^2120^	N^1869^-Q^2120^	N^1869^-Q^2120^	N^1865^-Q^2116^
3D	G^2121^-L^2588^	G^2121^-L^2588^	G^2121^-L^2588^	G^2117^-L^2584^

### Phylogenetic analyses

The phylogenetic trees constructed using the amino acid sequences of P1, VP1, P2 and P3 of the ERBV strains from this study and other erboviruses are shown in Fig. 1b, c, d and 1e respectively and the corresponding pairwise amino acid identities are shown in Table 2. For the trees constructed using P1 and VP1, strains 303, 396 and 421 were clustered with other strains of ERBV3, whereas strain 1228 was clustered with other strains of ERBV2, with high bootstrap supports. Correspondingly, these two regions of strains 303, 396 and 421 possessed significantly lower amino acid identities to the P1 and VP1 regions of ERBV1 and ERBV2 than to those of ERBV3 (Table 2). On the other hand, strain 1228 possessed significantly lower amino acid identities to the P1 and VP1 regions of ERBV1 and ERBV3 than to those of ERBV2 (Table 2). As for P2 and P3, the amino acid sequences of ERBV1, ERBV2 and the ERBV strains in the present study showed high identities of 96.3–97.7 % (Table 2), and therefore were indistinguishable from each other (Fig. 1).

### Genome analyses of ERBV3

Since no ERBV3 genome sequences were available and three (strains 303, 396 and 421) of the four strains were ERBV3, we further analyzed these three ERBV3 genomes and compared them with those of ERBV1 and ERBV2.

**Fig. 2:**
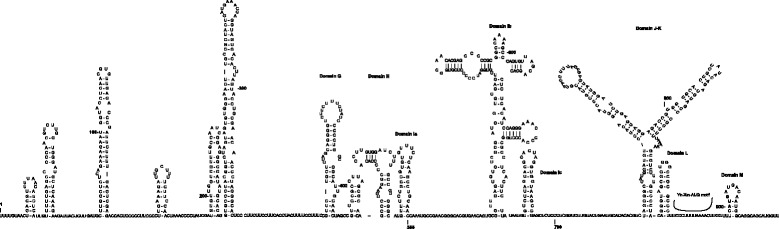
Secondary structure of predicted IRES in ERBV3 strain 303. The Yn-Xm-AUG motif is labeled

Similar to ERBV1 and ERBV2, the 5’ UTR of ERBV3 contained a polypyrimidine tract and 15 downstream stem-loop structures forming six domains (domains G to L) and demonstrated the conserved characteristics of a type II internal ribosome entry site (IRES) element. Domain G formed a stem-loop structure. Domains H, I, J, K and L are the main domains of the IRES element, responsible for directing the initiation of translation in a cap-independent manner, which requires both canonical translation initiation and IRES *trans*-acting factors [13]. Domain I was divided into sub-domains Ia, Ib and Ic. Domain Ib contained the characteristic tetra-loop conformation and conserved GNRA motifs. Upstream to the AUG start codon, the Yn-Xm-AUG motif was present at domain L (Fig. 2). Similar to other erboviruses, there was an L protein in the polyprotein of ERBV3 with putative protease activity.

The P1 (capsid-coding) regions in the genomes of ERBV3 encoded the capsid genes VP4, VP2, VP3 and VP1. Similar to ERBV1 and ERBV2, the cleavage sites at the junction of VP4/VP2, VP2/VP3 and VP3/VP1 in the three genomes of ERBV3 were Leu/Asp, Glu/Gly and Glu/Gly respectively. However, the cleavage sites of the three ERBV3 genomes at the cleavage junction of VP1/2A were Ser/Asn, whereas those of ERBV1 and ERBV2 as well as strain 1228 were Thr/Asn. Like ERBV1 and ERBV2, all the three ERBV3 genomes did not possess the [PS]ALXAXETG motif.

The P2 regions in the three ERBV3 genomes encoded non-structural proteins 2A, 2B and 2C. The 2A protein of picornaviruses is a highly variable region (9 to 305 amino acids). Similar to ERBV1 and ERBV2, the 2A proteins of ERBV3 was 16 amino acids in length and contained the Asn-Pro-Gly-Pro (NPGP) motif required for co-translational cleavage [14]. The 2A protein of ERBV3 exhibited 87.5–100 % amino acid identities to those of ERBV1 and ERBV2. The 2A protein shared 93.8 % amino acid identities among the three ERBV3 strains. Similar to ERBV1 and ERBV2, the 2A of ERBV3 did not possess the chymotrypsin-like structures with cysteine-reactive catalytic sites and the conserved GXCG motif found in the 2A proteinases of enteroviruses and rhinoviruses [14, 15]. The conserved H-box/NC motif, observed in members of the genera *Avihepatovirus*, *Kobuvirus*, *Tremovirus* and *Passerivirus* that was involved in cell proliferation control, was also absent [15–17]. Similar to most picornaviruses, the 2C of ERBV3 possessed the GXXGXGKS motif for NTP-binding [18] and DDLXQ for helicase activity [19].

The P3 regions in the three ERBV3 genomes encoded 3A, 3B (VPg, small genome-linked protein), 3C^pro^ (protease) and 3D^pol^ (RNA-dependent RNA polymerase). Similar to the 3C^pro^ of ERBV1 and ERBV2, the catalytic triad of the 3C^pro^ of ERBV3 was His-Asp-Cys. This was different from the 3C^pro^ of picornaviruses of some other genera, such as *Enterovirus*, *Sapelovirus* and *Kobuvirus*, which have catalytic triads of His-Glu-Cys. Similar to most picornaviruses, ERBV3 also contained the conserved GXCG and GXH motifs which form part of the active site of the protease. Similar to ERBV1 and ERBV2, ERBV3 did not possess the conserved RNA-binding motif KFRDI [20, 21]. Similar to other picornaviruses, the 3D^pol^ of ERBV3 contained the conserved KDE[LI]R, YGDD and FLKR motifs [22]. Similar to ERBV1 and ERBV2, ERBV3 possessed the GALPSG motif, instead of GG[LMN]PSG as in the genera *Aphthovirus*, *Cardiovirus*, *Enterovirus*, *Kobuvirus*, *Parechovirus*, *Sapelovirus*, *Senecavirus* and *Teschovirus*, in the 3D^pol^.

### Estimation of synonymous and non-synonymous substitution rates

Using the three ERBV3 genome sequences for analysis, the Ka/Ks ratios for the various coding regions were calculated (Table 4). All Ka/Ks ratios were all <0.1, suggesting that ERBV3 was stably evolving in horses.

**Table 4:** Synonymous and non-synonymous substitution rates of each coding region among the three genomes of ERBV

Putative proteins	No. of amino acids	Ka	Ks	Ka/Ks
L	219	0.0485	1.5837	0.0316
VP4	70–71	0.0146	1.6845	0.0074
VP2	256	0.0279	0.0000	-
VP3	229–230	0.0213	1.7612	0.0122
VP1	319–323	0.0792	2.2486	0.0363
2A	16	0.0692	1.3160	0.0728
2B	283	0.0233	1.4797	0.0177
2C	317	0.0106	0.9440	0.0113
3A	133	0.0231	0.9593	0.0242
3B	21	0.0294	0.6540	0.0639
3C	252	0.0126	0.6211	0.0203
3D	468	0.0038	0.2511	0.0152

### Estimation of divergence dates

**Fig. 3:**
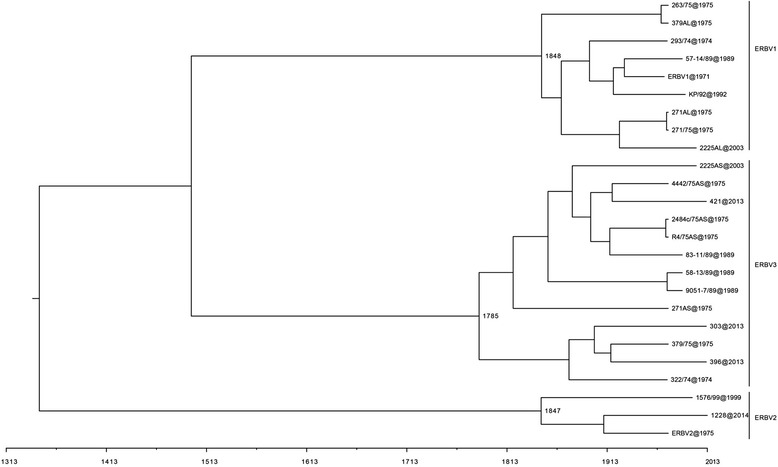
Estimation of tMRCA of ERBV1, ERBV2 and ERBV3 based on the VP1 gene. The mean estimated dates are labeled. The taxa are labeled with their sampling years

Using the uncorrelated lognormal distributed relaxed clock model (UCLD) [23] on VP1 gene, the date of the most recent common ancestor (MRCA) of ERBV3 was estimated to be 1785 (HPDs, 1176 to 1937), approximately 230 years before the present (Fig. 3). Moreover, the MRCA dates of ERBV1 and ERBV2 were estimated to be 1848 (HPDs, 1466 to 1949) and 1847 (HPDs, 1421 to 1963), approximately 167 and 168 years before the present, respectively (Fig. 3). The estimated mean substitution rate of the VP1 data set under the UCLD model was 2.09 × 10^−3^ substitution per site per year.

## Discussion

We report the first discovery of ERBVs in fecal samples of horses. Although both ERBV1 and ERBV2 have been found to be present in horses globally [24–29], so far ERBV3 has only been reported from horses in Australia, Japan and the United Kingdom [30–32] and no ERBVs have been detected in fecal samples. In the present study, ERBVs were observed in fecal samples of horses in Dubai. Phylogenetic analysis showed that one and three of the four strains were ERBV2 and ERBV3 respectively. Although the relatively higher chance of detecting ERBV3 in fecal samples may be related to its acid stability, the property does not exclude the presence of acid labile serotypes in stools. The observation of ERBVs in fecal samples of horses is analogous to infections by some other respiratory viruses, such as influenza virus, SARS coronavirus, rhinovirus and bocavirus, which have also been detected in the fecal samples of patients with infections by these viruses [10, 33–35]. For example, in our previous study on picornaviruses in fecal samples of children, human rhinovirus C was detected in four of 734 fecal samples from children with gastroenteritis [10]. Among these four children, three did not have any respiratory symptoms. Although detection of ERBVs in fecal samples by RT-PCR does not confirm infection of the gastrointestinal tract, such detection of ERBVs in fecal samples has direct implications for the infection control measures of infected horses. In addition to respiratory droplets, ERBVs may also spread through the feces of the horses. So far, the cellular receptors for ERBVs are unknown [36]. Further studies are required to examine if ERBVs could be associated with gastroenteritis or other gastrointestinal diseases in horses.

In addition to the first descriptions of ERBVs in equine fecal samples and ERBV3 in horses of the Middle East, this study also presents the first complete genome sequences of ERBV3. The genomes of ERBV1 and ERBV2 were sequenced in 1996 and 2001 respectively [11, 12]. On the other hand, only P1 sequences of ERBV3 were available. In this study, we sequenced three complete genomes of ERBV3. Similar to ERBV1 and ERBV2, ERBV3 also possesses a type II IRES element and most of the characteristic motifs in the various proteins encoded by the ERBV1 and ERBV2 genomes are also present in the ERBV3 genomes. The genome size of ERBV3 is comparable to those of ERBV1 and ERBV2, and collectively these genomes are some of the largest ones in the family *Picornaviridae*. The exceptionally large genome size of ERBV1, ERBV2 and ERBV3 is due to their long 5’-UTR and VP1. Their 5’-UTR are particularly long because of the characteristic presence of polypyrimidine tract upstream to their type II IRES. The low Ka/Ks ratio of all coding regions of the ERBV3 genomes showed that the virus is stably evolving in horses, supporting that horses are the natural reservoir of ERBV3. Since the P2 and P3 of ERBV1, ERBV2 and ERBV3 possess very high amino acid identities and are indistinguishable from each other, sequencing of P1, particularly its VP1 region, would be necessary for distinguishing among these three serotypes of ERBV.

**Fig. 4:**
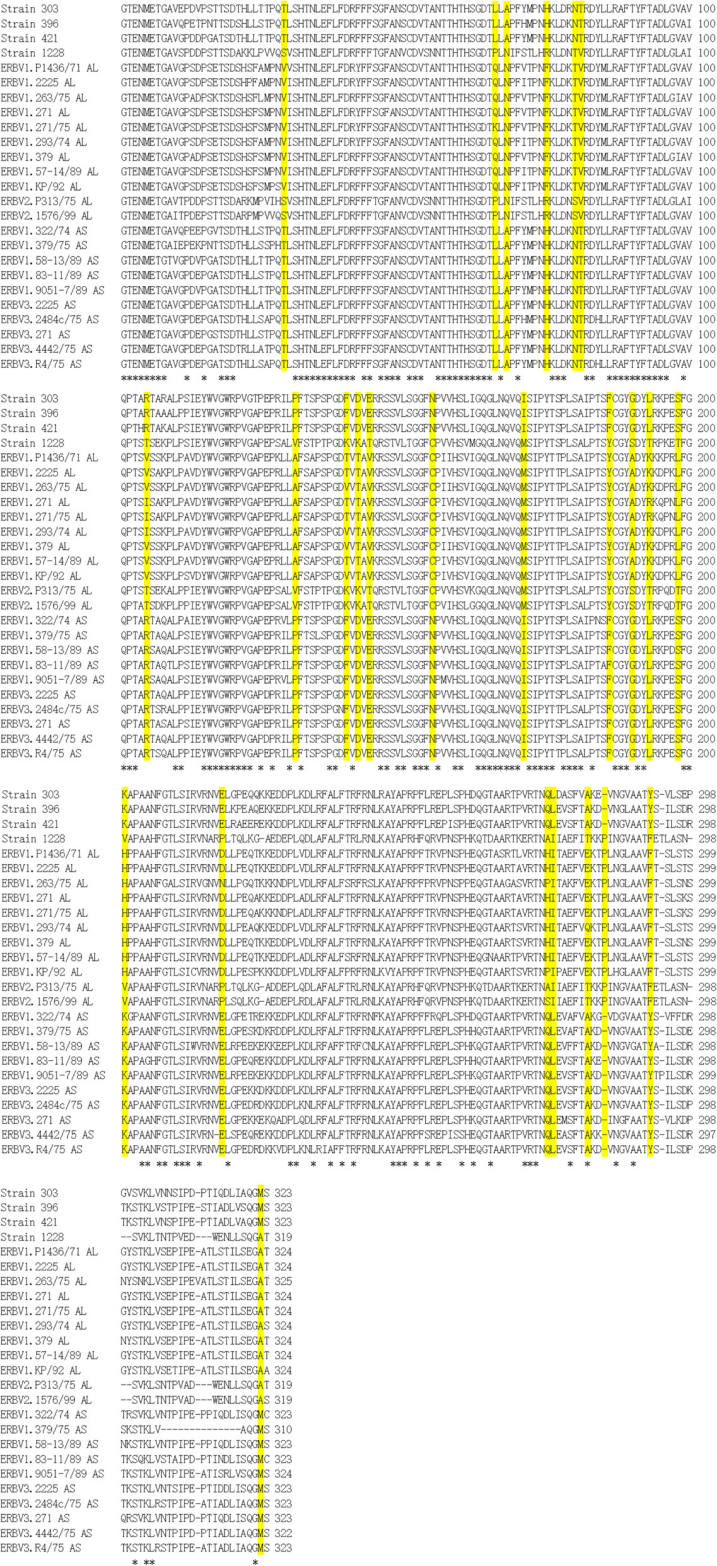
Multiple alignment of amino acid sequences from VP1 of 24 ERBVs. Positions at which ERBV3 is different from ERBV1 and ERBV2 strains are highlighted. Gaps introduced to maximize alignment are indicated by dashes. Conserved amino acids are indicated by an asterisk below the sequence alignment. Clustalw was used for the multiple alignments

The mechanism of acid stability in ERBVs remains to be determined. ERBV1 and ERBV2 are inherently acid labile, whereas ERBV3 is inherently acid stable and is able to survive at pH 3.3 [32]. Since it has been shown that the VP1 amino acid sequences of ERBV3 form a distinct cluster from those of ERBV1 and ERBV2 and the C-terminal of VP1 is the most exposed part of the virus, it is believed that the VP1 is responsible for acid stability of ERBV3 [37, 38]. In the present study, with the availability of the ERBV3 complete genomes, we confirmed the major difference between the genome sequences of ERBV3 and those of ERBV1 and ERBV2 lies in the amino acid sequences of their VP1 proteins. In a previous experiment, it has been shown that the acid stability of eight ERBV1 mutants induced by incubating an ERBV1 strain at lower pH can be increased and the viruses can survive at pH 4.0 but not pH 3.3 [31]. P1 sequencing of these eight ERBV1 mutants revealed a single nucleotide change at nucleotide position 901 (U → C) of VP1, leading to an amino acid change from tyrosine to histidine. In the genomes of ERBV3, this position is occupied by valine, arginine or lysine. In the ERBV2 genome from fecal sample in the present study as well as the only one ERBV2 genome from nasal sample, this amino acid at this position of their P1 sequences was not found (Fig. 4). Notably, there are other amino acid positions where the ERBV3 strains are different from the ERBV1 and ERBV2 strains (Fig. 4), although no mutagenesis studies have been carried out to determine their importance for acid stability. For example, position 201 are occupied by lysine in ERBV3 but valine and histidine in ERBV1 and ERBV2 respectively, and position 284 or 285 is occupied by proline in ERBV1 and ERBV2 but no amino acid is observed at this position in ERBV3 (Fig. 4). Therefore, it is still inconclusive on which amino acid(s) of the VP1 in ERBV3 are the most important for its acid stability and further experiments are still required to determine this.

## Conclusions

Both acid stable (ERBV3) and acid labile (ERBV2) ERBVs could be found in fecal samples of horses. Detection of ERBVs in fecal samples would have implications for their transmission and potential role in gastrointestinal diseases as well as fecal sampling as an alternative method of identifying infected horses.

## Methods

### Horse surveillance and sample collection

All equine fecal samples from Dubai were left-over specimens submitted for pathogens screening to Central Veterinary Research Laboratory in Dubai, United Arab Emirates (UAE) from April 2013 to July 2014 because of routine check-up, diarrhea or weight loss. A total of 29 fecal samples had been tested. All equine fecal samples from Hong Kong were collected from horses of The Hong Kong Jockey Club because of fever. A total of 47 fecal samples had been collected and tested from November 2010 to April 2011.

### RNA extraction

Viral RNA was extracted from the fecal samples using EZ1 Virus Mini Kit v2.0 (Qiagen, Hilden, Germany). The RNA was eluted in 60 μl of AVE buffer (Qiagen, Hilden, Germany) and was used as the template for RT-PCR.

### RT-PCR of 5’-untranslated region (UTR) of picornaviruses using conserved primers and DNA sequencing

Initial picornavirus screening was performed by amplifying a 111-bp fragment of the 5’-UTR of *Erbovirus* using primers (5’-GCTAAGGATGYCCTWCAGGT-3’ and 5’-GGCATAGAMGYTTTTTAAAC-3’) targeting conserved sequences of *Erbovirus*. Reverse transcription was performed using the SuperScript III kit (Invitrogen, San Diego, CA, USA) [17, 39–44] and the reaction mixture (10 μl) contained RNA, first-strand buffer (50 mM Tris–HCl pH 8.3, 75 mM KCl, 3 mM MgCl_2_), 5 mM DTT, 50 ng random hexamers, 500 μM of each dNTPs and 100 U Superscript III reverse transcriptase. The mixtures were incubated at 25 °C for 5 min, followed by 50 °C for 60 min and 70 °C for 15 min. The PCR mixture (25 μl) contained cDNA, PCR buffer (10 mM Tris–HCl pH 8.3, 50 mM KCl, 2 mM MgCl_2_ and 0.01 % gelatin), 200 μM of each dNTPs and 1.0 U *Taq* polymerase (Applied Biosystem, Foster City, CA, USA). The mixtures were amplified in 60 cycles of 94 °C for 1 min, 55 °C for 1 min and 72 °C for 1 min and a final extension at 72 °C for 10 min in an automated thermal cycler (Applied Biosystem, Foster City, CA, USA) [45–47]. Standard precautions were taken to avoid PCR contamination and no false-positive was observed in negative controls.

All PCR products were gel-purified using the QIAquick gel extraction kit (QIAgen, Hilden, Germany). Both strands of the PCR products were sequenced twice with an ABI Prism 3730*xl* DNA Analyzer (Applied Biosystems, Foster City, CA, USA), using the two PCR primers. The sequences of the PCR products were compared with known sequences of the 5’-UTR of picornaviruses in the GenBank database.

### Real-time quantitative RT-PCR

Real-time quantitative RT-PCR to detect the 3D^pol^ of ERBV was performed on the four positive fecal samples by the use of Premix Ex Taq™ (Probe qPCR) (TaKaRa, Japan) with primers 5’- TAATCAGCCACTGCCTCT-3’ and 5’-GAAACACAACGTCTGCCAA-3’ and probe 5’-6FAM-ATTACTCCAGCTGACAAGAGTTCCATCTTT-IBFQ-3’ and a LightCycler 96 System (Roche Applied Science, Mannheim, Germany). The reaction mixture contained 1x Premix Ex Taq (Probe qPCR), 0.3 μM of each primer, 0.1 μM of probe, 6.4 μl of nuclease free water, and 2 μl of cDNA template or standard. The cDNA template was generated as aforementioned. The reaction was subjected to thermal cycling at 95 °C for 30 s followed by 50 cycles of 95 °C for 5 s and 56 °C for 30 s.

### Genome sequencing

Four complete genomes of ERBVs (strains 303, 396, 421 and 1228), including the full 5’-UTR regions, were amplified and sequenced using strategies we previously used for complete genome sequencing of other picornaviruses, with the RNA extracted from the fecal samples as templates [17, 39, 40, 42–44, 48–52]. The RNA was converted to cDNA by a combined random-priming and oligo (dT) priming strategy. The cDNA was amplified by degenerate primers designed by multiple alignment of the genomes of ERBV1 and ERBV2 (GenBank accession no. NC_003983.1 and AF361253.1), and additional primers designed from the results of the first and subsequent rounds of sequencing. The 5’ ends of the viral genomes were confirmed by rapid amplification of cDNA ends using the SMARTer RACE cDNA Amplification Kit (Clontech, USA). Sequences were checked manually and assembled to produce final sequences of the full viral genomes.

### Genome analysis

Nucleotide sequences of the genomes and deduced amino acid sequences of the encoded polyproteins were compared to those of other picornaviruses. Unrooted phylogenetic tree of 5’-UTR was constructed using neighbor-joining method for aligned nucleotide sequences in ClustalX 2.1. Maximum-likelihood phylogenetic trees of P1, VP1, P2 and P3 were constructed using PhyML 3.0 program [53] and Approximate Likelihood-Ratio Test (aLRT) method [54]. Secondary structure prediction in the 5’-UTR was performed using RNAstructure Web Server on strain 303 [55].

### Estimation of synonymous and non-synonymous substitution rates

The number of synonymous substitutions per synonymous site, Ks, and the number of non-synonymous substitutions per non-synonymous site, Ka, for each coding region among the three strains of ERBV3 were calculated using KaKs_Calculator 2.0 [56].

### Estimation of divergence dates

The tMRCA was estimated based on an alignment of VP1 sequences, using the UCLD in BEAST version 1.8 (http://beast.bio.ed.ac.uk/) [23]. Sampling dates of all strains were collected from the literature or from the present study, and were used as calibration points. Sequences were aligned according to the codon positions. Depending on the data set, Markov chain Monte Carlo (MCMC) sample chains were run for 2 × 10^7^ states, sampling every 1000 generations under the HKY/SRD06 model of substitution. A constant population coalescent prior was assumed for all data sets. The median and HPD were calculated for each of these parameters from four identical but independent MCMC chains using TRACER 1.3 (http://beast.bio.ed.ac.uk). The tree was annotated by TreeAnnotator, a program of BEAST and displayed by FigTree (http://tree.bio.ed.ac.uk/software/figtree/).
